# Abnormal Functional Connectivity Density in Amyotrophic Lateral Sclerosis

**DOI:** 10.3389/fnagi.2018.00215

**Published:** 2018-07-17

**Authors:** Weina Li, Jiuquan Zhang, Chaoyang Zhou, Wensheng Hou, Jun Hu, Hua Feng, Xiaolin Zheng

**Affiliations:** ^1^Key Laboratory of Biorheological Science and Technology, Ministry of Education, Bioengineering College, Chongqing University, Chongqing, China; ^2^Department of Neurosurgery, Southwest Hospital, Third Military Medical University, Army Medical University, Chongqing, China; ^3^Chongqing Collaborative Innovation Center for Brain Science, Chongqing, China; ^4^Department of Radiology, Chongqing University Cancer Hospital, Chongqing, China; ^5^Department of Neurology, Southwest Hospital, Third Military Medical University, Army Medical University, Chongqing, China

**Keywords:** amyotrophic lateral sclerosis, functional connectivity density (FCD), functional magnetic resonance imaging, resting state, functional connectivity

## Abstract

**Purpose:** Amyotrophic lateral sclerosis (ALS) is a motor neuro-degenerative disorder that also damages extra-motor neural pathways. A significant proportion of existing evidence describe alterations in the strengths of functional connectivity, whereas the changes in the density of these functional connections have not been explored. Therefore, our study seeks to identify ALS-induced alternations in the resting-state functional connectivity density (FCD).

**Methods:** Two groups comprising of 38 ALS patients and 35 healthy participants (age and gender matched) were subjected to the resting-state functional magnetic resonance imaging (MRI) scanning. An ultra-fast graph theory method known as FCD mapping was utilized to calculate the voxel-wise short- and long-range FCD values of the brain for each participant. FCD values of patients and controls were compared based on voxels in order to discern cerebral regions that possessed significant FCD alterations. For areas demonstrating a group effect of atypical FCD in ALS, seed-based functional connectivity analysis was then investigated. Partial correlation analyses were carried out between aberrant FCDs and several clinical variables, controlling for age, gender, and total intracranial volume.

**Results:** Patients with ALS were found to have decreased short-range FCD in the primary motor cortex and increased long-range FCD in the premotor cortex. Extra-motor areas that also displayed extensive FCD alterations encompassed the temporal cortex, insula, cingulate gyrus, occipital cortex, and inferior parietal lobule. Seed-based correlation analysis further demonstrated that these regions also possessed disrupted functional connectivity. However, no significant correlations were identified between aberrant FCDs and clinical variables.

**Conclusion:** FCD changes in the regions identified represent communication deficits and impaired functional brain dynamics, which might underlie the motor, motor control, language, visuoperceptual and high-order cognitive deficits in ALS. These findings support the fact that ALS is a disorder affecting multiple systems. We gain a deeper insight of the neural mechanisms underlying ALS.

## Introduction

Amyotrophic lateral sclerosis (ALS) is a progressive neuro-degenerative disease that involves degeneration of both upper and lower motor neurons. This often fatal condition is marked by muscular paralysis, spasticity, and bulbar signs. Despite the predominant deterioration of the motor system in ALS, extensive evidence has documented several non-motor symptoms such as language impairments, visuoperceptual deficits, and cognitive deterioration (Verma et al., 2013; Stoppel et al., 2014; Ash et al., 2015; Loewe et al., 2017). Extensive neuro-imaging and neuro-pathological studies have supplied irrefutable evidence of the complexity of ALS, demonstrating that functional and anatomical cerebral lesions are present not only in the precentral cortices and corticospinal tracts, but also in the frontal, temporal, visual processing cortices, cerebellum, and corpus callosum (Chio et al., 2014; Turner and Verstraete, 2015; Grolez et al., 2016).

The study of brain pathologies in various clinical cohorts have seen the rise of the use of resting-state functional magnetic resonance imaging (fMRI), given its ease of application and ability to characterize intricate cerebral circuits. Resting-state functional connectivity is a study of functional interactions between spatially remote regions, which quantifies the temporal correlations of inter-regional spontaneous fluctuations in cerebral activity. The presence of widespread reorganization of functional connectivity has been documented in ALS patients. Studies that have investigated resting-state cerebral functional connectivity in patients with ALS concur in their findings of the attenuated functional connectivity in the sensorimotor circuits (Mohammadi et al., 2009; Zhou et al., 2013; Chenji et al., 2016; Fang et al., 2016) and in behavioral and cognition-related brain networks (Luo et al., 2012; Agosta et al., 2013; Zhou et al., 2016; Loewe et al., 2017), both of which are in line with the altered structural connectivity in motor and extra-motor systems. Conversely, studies have also demonstrated elevated functional activity within and beyond the motor and premotor cortex, despite reduced structural connection (Agosta et al., 2011; Douaud et al., 2011; Zhou et al., 2016), or a mixed picture of both decreased and increased functional coherence within cortical sub-regions (Zhou et al., 2014). However, all of these functional connectivity studies were based on independent component analysis (ICA) or seed-based analysis. Each of these techniques have their own pitfalls; ICA is well suited for the measurement of global cerebral connectivity and may not be as specific for local measures of connectivity, while the seed-based analysis relies heavily on pre-selection of seed regions and there is a risk of under-representation of the complex nature of cerebral inter-circuit relationships.

Functional connectivity density mapping (FCDM), an ultra-fast data driven method, quantifies functional connections between a given voxel and all the other voxels in the entire brain (Tomasi and Volkow, 2010). Voxels that possess a higher number of functional connections to other brain voxels have greater FCD values, suggesting that these voxels may be more crucial for processing information. Both short- and long-range FCDs can be derived from global FCD scores, which are in turn based on relationships between neighboring voxels (Tomasi and Volkow, 2012). It has been demonstrated that FCDM estimates are comparable to prior estimates of cortical hubs (Tomasi and Volkow, 2011; Tomasi et al., 2013), and are resilient to the effects of temporal dynamics and robustness at high spatiotemporal dynamics in large samples of multicenter (Tomasi et al., 2016a,b; Cohen et al., 2017). Independent laboratories have also shown disrupted FCD in attention deficit hyperactivity disorder (ADHD); Tomasi and Volkow, 2012), non-epileptic seizures (Ding et al., 2014), schizophrenia (Tomasi and Volkow, 2014; Zhuo et al., 2014; Wang et al., 2017), traumatic axonal injury (Caeyenberghs et al., 2015) cocaine addiction (Konova et al., 2015), and congenital blindness (Qin et al., 2015).

Unlike the functional connectivity analysis, which is a one-to-one measurement in that it only quantifies the strength of functional connections between two seeds or two voxels, the FCDM measures functional connectivity density (FCD) of voxels and reflects one-to-more relationship (Zhuo et al., 2014). Thus, investigating the changes of FCD in ALS may provide us with the knowledge of the changes of the communication capacity of voxels and functional brain dynamics in information processing. This may enlighten our understanding of the neural mechanisms of ALS from a new perspective.

The current study evaluates resting-state fMRI-derived FCD maps in both ALS patients and healthy controls (HCs) in order to determine FCD dysfunctions in ALS. Regions that showed a group effect of atypical FCD in ALS were then subjected to more detailed analysis where their connections to other regions were scrutinized to determine which ones possessed deficits as alluded by the initial FCD. Correlation analysis was performed to further explore potential associations between clinical variables and the mean FCD of each region with significant group differences.

## Materials and Methods

### Subjects

Thirty-eight patients (25 males/13 females) that were first diagnosed as probable or definite sporadic ALS based on the revised El Escorial criteria (Brooks et al., 2000) were enrolled during their visits to the Department of Neurology at Southwest Hospital in Chongqing, China. An “ALS Functional Rating Scale-Revised” (ALSFRS-R) score was assigned to each patient (Cedarbaum et al., 1999) within 12 h after initial magnetic resonance imaging (MRI). Disease duration in these patients were defined as the duration between symptom onset to initial MRI scan while the rate of disease progression was defined as (48-ALSFRS-R)/(disease duration) (Kimura et al., 2006). None of the patients in this study was received any form of therapeutic interventions. Exclusion criteria were: (1) cognitive impairment (Montreal Cognitive Assessment score ≤ 26); Nasreddine et al., 2005); (2) clinical diagnosis of frontotemporal dementia (Neary et al., 1998); (3) family history of motor system diseases; and (4) presence of other major systemic, psychiatric, or neurological illnesses. Thirty-five HCs ( 21 males/14 females) were recruited from the local population. All controls had normal brain MRIs and were negative for a history of neurological or psychiatric conditions. The Endinburg inventory confirmed that all participants were right-handed (Cedarbaum et al., 1999).

This study was carried out ethically in compliance to recommendations set by the International Ethical Guidelines on Biomedical Research Involving Human Subjects. Study protocols were evaluated and approved by the Medical Research Ethics Committee of the Southwest Hospital. All subjects provided written informed consent, in accordance to the Helsinki Declaration.

### MRI Acquisition

A 3.0-T Siemens Tim Trio whole-body MRI system (Siemens Medical Solutions, Erlangen, Germany) was utilized to acquire all functional images. During data collection, participants were told to close their eyes, rest, to empty their mind of thought and to stay awake. Transverse imaging data were acquired with an echo-planar imaging (EPI) sequence using the following settings: TR = 2,000 ms, TE = 30 ms, flip angle = 90°, slices = 36, thickness = 3 mm, slice gap = 1 mm, FOV = 192 mm × 192 mm, in-plane matrix = 64 × 64, and voxel size = 3.0 mm × 3.0 mm × 3.0 mm. A total of 240 volumes that resulted in a 480 s scan time were collected for each subject. Sagittal oriented 3D T1-weighted anatomical images were obtained using the following volumetric 3D magnetization-prepared rapid gradient–echo (MP-RAGE) sequence (TR = 1,900 ms, TE = 2.52 ms, flip angle = 9°, slices = 176, slice thickness = 1 mm, FOV = 256 mm × 256 mm, matrix size = 256 × 256, and voxel size = 1 mm × 1 mm × 1 mm) on each subject.

### Data Preprocessing

The SPM8^1^ software was used to preprocess the resting-state fMRI data. In all participants, 10 initial volumes were removed from analysis to allow participants to adapt to machine noise and to allow brain signals to equilibrate. The remaining volumes were then subjected to slice time correction, realignment, coregistration, spatially normalized with the Montreal Neurological Institute (MNI) EPI template (using unified segmentation on T1 image) and re-sampled to 3 mm cubic voxels. None of the subjects moved more than 2° rotation or 2 mm translation. Micro head movement parameter—mean frame-wise displacement (mFD) was also assessed for each participant, which indexes the volume-to-volume changes in head position (Jenkinson et al., 2002). Three participants (including two ALS patients and one HC) were excluded because they had a mFD higher than group mean of mFD plus two times the standard deviation of mFD (Yan et al., 2013). After normalization, the nuisance variables, including the average BOLD signals of the ventricular and white matter, six head motion parameters as well as the linear drift signals were linearly regressed out from further analysis. Finally, the fMRI data were subjected to a 0.01–0.08 Hz band-pass filtration.

### FCD Mapping

The FCD map of each participant was generated with a neuroscience information toolbox (NIT, version1.1^2^) derived from a protocol proposed by Tomasi and Volkow (2010). A gray matter mask was predefined by including tissue with probabilities more than 20% of the gray matter probability template in SPM8 to restrict the FCD calculations (Zuo et al., 2012; Zhou et al., 2016). The quantity of functional connections, *K* (*x*_0_), was defined based on Pearson correlations of time-varying signals at x_0_ and those at other voxels using a threshold of *R* > 0.6, selected based on prior studies (Tomasi and Volkow, 2010,Tomasi and Volkow, 2012,Tomasi and Volkow, 2012,Tomasi and Volkow, 2012; Caeyenberghs et al., 2015). The total number of voxels functionally connected to any given voxel (*x*_0_), also known as “degree,” represented its global FCD value. The short-range FCD at each voxel in the brain was calculated with a “growing algorithm,” and represented the sum of all the elements in its immediate functional connectivity cluster vicinity. Briefly, a voxel (*x*_j_) could be added to the list of functionally connected voxels with *x*_0_ only if it was adjacent to a voxel that was linked to *x*_0_ by a continuous path of functionally connected voxels and *R*_0j_ > 0.6 (Tomasi and Volkow, 2010). This formula was applied in an iterative manner to all adjacent voxels listed in the group of voxels functionally connected to *x*_0_ until no new voxels can be added to the list. Long-range FCD at *x*_0_ was derived from the subtraction of the short-range FCD from the global FCD to isolate the number of distal functional connections. Information garnered from short- and long-range FCDs pertain to the relative voxel distance instead of their absolute spatial distance. All FCD maps were then rescaled to the individual average FCD of the whole brain [FCD_rescaled_ (*x*, *y*, *z*) = FCD (*x*, *y*, *z*)/mean (FCD)] to normalize the FCD distributions and reduce the effect of person-to-person variability. Finally, a 6 mm Gaussian kernel was used to spatially smooth the FCD maps prior to group-level analysis.

## Statistical Analysis

Statistical analyses that were not related to voxel computations were carried out with SPSS (SPSS Statistics for Windows, V.19.0. Chicago, IL, United States). Continuous data normality within sub-groups was assessed by the Shapiro–Wilk test. Differences between ALS patients and HCs regarding demographical data (i.e., age, gender), micro head movement parameter (i.e., mFD), and total intracranial volume [TIV, derived from the summation of the volumes of gray matter, white matter and cerebrospinal fluid, all of which were obtained during segmentation in the preprocessing step (Ridgway et al., 2011)] were calculated as follows: continuous, normally distributed variables were subjected to the two-sample *t* -test ; continuous, non-normally distributed variables were analyzed with the Mann–Whitney *U-* test while the chi-square test was used for categorical variables. The significance threshold was set at *p* < 0.05 for all analyses.

The RESTplus software^3^ was used to perform voxel-wise two-sample *t* -test of FCD maps between ALS patients and HCs. All comparisons were subjected to a voxel-by-voxel GLM with age, gender and TIV as covariates, independently for short- and long-range FCDs. A Monte Carlo simulation was used to correct for multiple comparisons (AlphaSim program, parameters including: single voxel *p* = 0.01, 5,000 simulations, cluster connection radius *r* = 5 mm, full width at half maximum = 6 mm, with a gray matter mask and a resolution of 3 mm × 3 mm × 3 mm), which resulted a corrected threshold of *p* < 0.05.

A seed-based functional connectivity analysis was performed to show the FCD results in the context of common functional connectivity analysis. Brain regions with significant alterations of FCD were chosen as seeds. Pearson correlation coefficients were calculated between the averaged time series of the chosen seeds and those of other voxels in the gray matter regions of the brain, respectively. The correlation coefficients maps were subjected to Fisher’s *r* -to -*z* transformation to improve normality, and the resultant *z* maps were spatially smoothed (FWHM = 6 mm) as in FCD processing. A voxel-wise two-sample *t* -test was used to carry out group comparison and a corrected *p* -value of 0.05 was taken as the statistical threshold (Alphasim corrected). Both the chosen seeds and the group comparison results of seed-based functional connectivity would be described based on subregions of the Brainnetome atlas, which is constructed based on both structural and connectivity features, and possesses finer-grained brain subregions (210 cortical subregions, 36 subcortical subregions, and 28 cerebellar subregions; Fan et al., 2016; and Supplementary File of Fan’s paper). This atlas was deemed to be more suitable than a conventional brain atlas (Brainnetome atlas;^4^ Supplementary Table S1 showed detailed descriptions of the atlas subregions).

The mean FCD of each cluster that possessed significant group differences were extracted for each patient. Several clinical parameters (i.e., rate of disease progression, disease duration and ALSFRS-R scores), were then tested using partial correlation analysis to determine if any of them were associated to those FCD values. Age, gender, and TIV served as control variables. The Bonferroni method was used to carry out multiple comparison correlations (*p* < 0.05).

We also controlled for the confounding effect of micro head movement, “mFD” in this paper, in addition to gender, age and TIV in all the group comparisons and correlation analysis process, as we could not rule out the possibility that results were still sensitive to gross head motion effects event after the regression of six head motion parameters during data preprocessing (Van Dijk et al., 2012; Tomasi and Volkow, 2014; Qin et al., 2015). Furthermore, given that global signal regression (GSR) is commonly utilized as a control for scanner instabilities in studies of resting-state functional connectivity (Chen et al., 2012), we performed additional investigations of the effect of GSR on the comparisons of short- and long-range FCD between HCs and ALS patients.

## Results

### Demographic and Clinical Data

**Table 1** depicts a summary of all demographic and clinical data of our cohort. There were no significant variations between both ALS and HC groups in terms of age, gender, TIV, and mFD.

**Table 1:** Demographic and clinical data of the participants.

	Patients	Controls	Test stats (*p*)
Age (years)	48.42 ± 9.26	49.03 ± 11.37	0.81^a^
Male/female	23/13	21/13	0.85^b^
Mean frame-wise displacement	0.10 (0.05)^d^	0.11 (0.07)^d^	0.79^c^
TIV	1.596 ± 0.16	1.642 ± 0.16	0.24^a^
El Escorial criteria (probable/definite)	22/14	/	/
Disease duration (months)	22.89 ± 18.06	/	/
ALSFRS-R	30.44 ± 6.77	/	/
Disease progression rate	1.17 ± 0.90	/	/


*TIV, total intracranial volume; ALSFRS-R, ALS functional rating scale-revised. ^*a*^Two-sample t-test. ^*b*^Pearson’s chi-square test. ^*c*^Mann–Whitney U-test. ^*d*^Median (interquartile range).*

### Differences in Short-Range FCD Between ALS Patients and HCs

**FIGURE 1:**
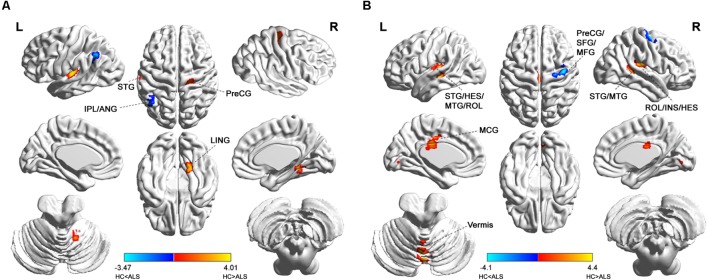
Distribution of statistical differences in short-range and long-range FCDs between ALS patients and healthy controls (HCs). Voxel-wise two-sample *t* -test with three covariates (age, gender, total intracranial volume) was used to contrast short-range and long-range FCD maps across groups (*p* < 0.05, AlphaSim corrected) in no global signal regression condition. **(A,B)** shows the distribution of short-range FCD and long-range FCD differences between the two groups, respectively, with warm color representing FCD value of HC lager than ALS and cold color denoting FCD value of HC smaller than ALS. ANG, angular gyrus; HES, heschl gyrus; INS, insula; IPL, inferior parietal; LING, lingual gyrus; MCG, middle cingulate; MFG, middle frontal gyrus; MTG, middle temporal gyrus; PreCG, precental gyrus; ROL, rolandic operculum; SFG, superior frontal gyrus; STG, superior temporal gyrus; L, left; R, right.

Group comparisons showed that the ALS patients were found to have markedly attenuated short-range FCDs in the right precentral gyrus [Brodmann area (BA) 4; Brainnetome #60, right lingual gyrus (BA19; Brainnetome #196), and left superior temporal gyrus (BA41; Brainnetome #73, 71) but had markedly elevated short-range FCDs in the left angular gyrus and the left inferior parietal lobule (BA39, 40; Brainnetome #141, 137; **Figure 1A** and **Table 2**).

**Table 2:** Short- and long-range FCD alterations in ALS patients compared with healthy controls^a^.

FCD	Cluster index	Brain regions	Brodmann’s area	Brainnetome atlas subregion^b^	Cluster size (voxels)^c^	Peak MNI coordinate (*x*, *y*, *z*)	Peak intensity (*t-* value)
Short-range FCD		**HC > ALS**					
	1	Right lingual gyrus	19	rLinG (Brainnetome #196)	37	18, -51, -12	3.2628
	2	Left superior temporal gyrus	41	TE1.0 and TE1.2 (Brainnetome #73), A41/42 (Brainnetome #71)	62	-54, -21, 6	4.011
	3	Right precentral gyrus	4	A4t (Brainnetome #60)	60	24, -24, 51	3.3177
		**HC < ALS**					
	4	Left inferior parietal lobule, left angular gyrus	40, 39	A40c (Brainnetome #141), A39rd (Brainnetome #137)	48	-45, -54, 42	-3.4677
Long-range FCD		**HC > ALS**					
	5	Vermis VI	/	Cerebellum_Vermis_VI (Brainnetome #252)	65	0,-69,-6	4.4033
	6	Left superior temporal gyrus, left heschl gyrus, left middle temporal gyrus, left rolandic operculum	41, 22	TE1.0 and TE1.2 (Brainnetome #73), A41/42 (Brainnetome #71), aSTS (Brainnetome #87), A22c (Brainnetome #75)	172	-45, -27, 0	4.154
	7	Right superior temporal gyrus, right middle temporal gyrus	21, 22, 42	rpSTS (Brainnetome #122), TS (Brainnetome #124)	41	54, -42, 12	3.5023
	8	Right rolandic operculum, right insula, right heschl gyrus	13	A40rv (Brainnetome #146), G (Brainnetome #164)	87	36, -24, 18	3.9736
	9	Left middle cingulate gyrus	23	A23c (Brainnetome #185), A23d (Brainnetome #175)	63	-6, -24, 27	3.7521
		**HC < ALS**					
	10	Right precentral gyrus, right superior frontal gyrus, right middle frontal gyrus	6	A6cdl (Brainnetome #56), A4ul (Brainnetome #58)	67	39, -9, 63	-4.0984


*^*a*^This result was computed in no global signal regression condition. Supplementary Table S1 was obtained in global signal regression condition. ^*b*^Brainnetome Atlas, a brain atlas based on connectional architecture*, http://atlas.brainnetome.org. *^*c*^Statistical significance was set at a voxel-wise p < 0.01, in conjunction with cluster wise AlphaSim (rmm = 5, clusters = 36) to correct for multiple comparisons.*

### Differences in Long-Range FCD Between ALS Patients and HCs

Patients with ALS showed decreased long-range FCD in the bilateral superior and middle temporal gyrus, bilateral heschl gyrus, bilateral rolandic operculum, and right insula (BA41, 42, 22, 21, 13; Brainnetome #73, 71, 87, 75, 122, 124, 146, 164), and also in cerebellar vermis VI (Brainnetome #252), left middle cingulate gyrus (BA23; Brainnetome #185, 175). In addition, ALS patients also had heightened long-range FCDs in the right precentral gyrus, right superior and middle frontal gyrus (BA6, Brainnetome #56, 58; **Figure 1B** and **Table 2**).

### Seed-Based Functional Connectivity

**FIGURE 2:**
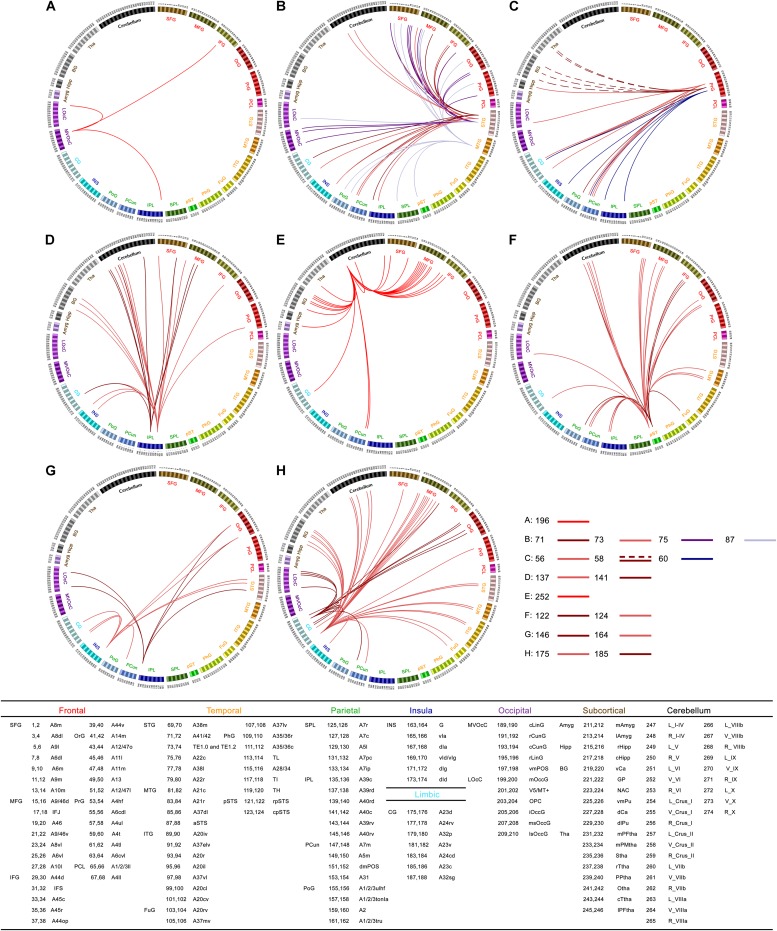
Statistical difference of seed-based functional connectivity for the abnormal FCD regions between ALS patients and healthy controls (HCs): connectograms. Between-group differences in seed-based functional connectivity analyses with seeds belonging to right rostral lingual gyrus [cluster 1, Brainnetome #196 **(A)**], left superior and middle temporal gyrus [cluster 2 and cluster 6, Brainnetome #71, 73, 75, 87 **(B)**], right precentral gyrus [cluster 3 and cluster 10, Brainnetome #60, and Brainnetome #56, 58 **(C)**], left inferior parietal lobule [cluster 4, Brainnetome #137, 141 **(D)**], cerebellar vermis VI [cluster 5, Brainnetome #252 **(E)**], right superior temporal sulcus [cluster 7, Brainnetome #122, 124 **(F)**], right rostroventral of BA40 and right insula gyrus [cluster 8, Brainnetome #146, 164 **(G)**], left middle cingulate gyrus [cluster 9, Brainnetome #175, 185 **(H)**], respectively. In the connectograms, each circular segment corresponds to a subregion from brannetome atlas (Brainnetome atlas: http://atlas.brainnetome.org ; Supplementary Table S1 showed detailed descriptions of atlas subregions). ALS-related seed-based functional connectivity changes are illustrated at the level of subregion pairs. A link connecting two subregions indicates significantly decreased (solid lines) or increased (dashed lines) functional connectivity in ALS (*p* < 0.05, Alphasim corrected).

To present FCD results in the context of common functional connectivity analysis, we chose the clusters with significant alterations of FCD as seeds of seed-based functional connectivity analysis. For cluster 1, right rostral lingual gyrus (Brainnetome #196), we found decreased functional connectivity in inferior frontal gyrus, lateral occipital cortex, and inferior parietal lobule in ALS (**Figure 2A**). For cluster 2 and cluster 6, left superior and middle temporal gyrus (Brainnetome #71, 73, 75, 87), we observed common decreased functional connectivity in frontal lobe (superior, middle, inferior and orbital frontal gyrus, precentral gyrus), occipital lobe (medio ventral and lateral occipital cortex), parietal lobe (superior and inferior parietal lobule, precuneus, and postcentral gyrus), and also in posterior superior temporal sulcus, insular gyrus, cingulate gyrus, cerebellar vermis VI and right Crus I in ALS patients (**Figure 2B**). For cluster 3, right precentral gyrus (trunk region of BA4, Brainnetome #60), we found decreased functional connectivity in inferior parietal lobule, precuneus, cingulate gyrus and insula in patients group (**Figure 2C**). For cluster 4, left inferior parietal lobule (Brainnetome #137, 141), we found decreased functional connectivity in cerebellum, frontal lobe (superior, middle and inferior frontal gyrus, and precentral gyrus), basal ganglia area (globus pallidus, ventromedial putamen, dorsal caudate), cingulate gyrus and insula, but no significant increased functional connectivity was found in ALS patients (**Figure 2D**).

For cluster 5, cerebellar vermis VI (Brainnetome #252), decreased functional connectivity was found mainly in middle, inferior and superior frontal gyrus, basal ganglia, and also in precuneus, thalamus, amygdala, cingulate gyrus, and other parts of cerebellum (right cerebellum X and left Crus II) in ALS patients (**Figure 2E**). For cluster 7, right superior temporal sulcus (Brainnetome #122, 124), decreased functional connectivity was found in frontal lobe (superior, middle, inferior, and orbital frontal gyrus), parietal lobe (angular, precuneus, and postcentral gyrus), cerebellum, middle temporal gyrus, fusiform, cingulate gyrus, and lateral occipital cortex in patients (**Figure 2F**). For cluster 8, right rostroventral of BA40 (Brainnetome #146) and right insula gyrus (Brainnetome #164), decreased functional connectivity was found in orbital frontal gyrus, superior temporal gyrus, middle and medial superior occipital gyrus, cingulate gyrus and precuneus in patients group (**Figure 2G**). For cluster 9, left middle cingulate gyrus (left dorsal and caudodorsal of BA23, Brainnetome #175, 185), we found decreased functional connectivity mainly in middle frontal gyrus, cerebellum, basal ganglia, temporal lobe (superior, middle and inferior temporal gyrus, fusiform gyrus), and also in orbital frontal gyrus, lateral occipital cortex, precuneus, and insula in ALS (**Figure 2H**). For cluster 10, right precentral gyrus (caudal dorsolateral of BA6 and upper limb region of BA4, Brainnetome #56, 58), we observed increased functional connectivity in right caudate and bilateral thalamus, and decreased connectivity precuneus, postcentral gyrus, insula, cingulate gyrus, lateral occipital cortex, and caudal hippocampus in ALS patients (**Figure 2C**).

### Relationship Between FCD and Clinical Parameters in ALS Patients

There were no statistically significant correlations between the aberrant short- or long-range FCDs and clinical parameters under the controlling for age, gender and TIV after Bonferroni correction.

### Other Conditions

In addition to controlling for age, gender and TIV, we also controlled for micro head movement (“mFD” in this paper) as covariate regarding its potential confounding effects. We found that additional controlling for mFD during statistical analyses of short-range and long-range FCDs between HCs and ALS patients yielded almost the same results to analyses that did not control for mFD, with the exception of slight differences in cluster size and peak intensity (Supplementary Figure S1). No abnormal FCDs were found to correlate with clinical variables with the additional controlling for mFD after Bonferroni correction either.

Group comparisons of FCDs with GSR showed similar results to group comparisons without GSR, besides that significantly decreased FCDs (both short-range and long-range) were observed in the bilateral thalamus (Brainnetome #238, 237) and significantly increased FCDs (both short-range and long-range) were found in the right medial superior frontal gyrus (BA8, Brainnetome #6) in ALS patients compared with HCs (Supplementary Figure S2 and Supplementary Table S2).

## Discussion

Our investigations utilized a data-driven voxel-based method in studying the map changes of short- and long-range FCD in patients with ALS. Unsurprisingly, we found abnormal FCDs in both primary motor cortex and premotor cortex in ALS patients, given that it is primarily a motor neuron disease. Interestingly, we also observed extensive FCD alterations in the temporal cortex, insula, cingulate gyrus, occipital cortex, and inferior parietal lobule, which is in accordance to existing literature that highlights ALS as more than just a motor disorder involving widespread dysfunction in non-motor cerebral regions. As revealed by seed-based correlation analysis, disrupted functional connectivity was also found in these regions, which helped to interpret the changes of FCD in ALS patients. The FCD changes allude to disruptions in functional brain dynamics and dysfunctional communication capabilities in these areas of the brain, providing novel insights into the underlying neural circuitry of ALS.

### Changes of FCD in Motor Cortex

Alteration of the motor cortex is the most prominent neuroimaging feature in ALS (Cosottini et al., 2012; Foerster et al., 2013; Chio et al., 2014; Menke et al., 2017). Focusing on the motor system, decreased short-range FCD was observed in the primary motor cortex (right precentral gyrus, BA4), whereas the premotor cortex (right precentral gyrus/superior and middle frontal gyrus, BA6) was found to have elevated long-rate FCD. This pattern of changes is in line with the decreased and increased functional activities and functional connections in these regions documented in prior studies (Mohammadi et al., 2009; Jelsone-Swain et al., 2010; Agosta et al., 2011; Douaud et al., 2011; Tedeschi et al., 2012; Zhou et al., 2013).

While performing a voxel-wise resting graph theory-based network study, Zhou et al. (2016) found decreased degree centrality present in bilateral primary motor and sensory motor cortices in ALS patients. Studies replicating this protocol have also demonstrated significantly reduced sensorimotor network functional connectivity in ALS patients (Mohammadi et al., 2009; Zhou et al., 2013; Chenji et al., 2016; Fang et al., 2016). In keeping with these previous works, we observed reduced functional connectivity between motor cortex and the precuneus, cingulate gyrus, inferior parietal lobule, postcentral gyrus, insula, lateral occipital cortex, and caudal hippocampus in seed-based functional connectivity analysis (**Figure 2C**), which may suggest a disease-related functional deprivation. Likewise, loss of neurons in the motor cortex as evidenced by reduced cortical thickness, gray matter volume, degenerating fibers and attenuations in *N* -acetylaspartate :creatine (NAA:Cr) ratio or NAA levels (suggesting neuronal degeneration) when measured by structural imaging, PET and ^1^H-magnetic resonance spectroscopy (MRS) have been demonstrated in previous studies (Verstraete et al., 2012; Trojsi et al., 2013; Verma et al., 2013; Chio et al., 2014; Zhu et al., 2015; Maani et al., 2016; Shen et al., 2016; Buhour et al., 2017).

Interestingly, elevated cerebral functional activity was also noted in the motor and premotor cortices as well as other regions in spite of the disrupted structural connections (Agosta et al., 2011; Douaud et al., 2011). The results of our experiments are quite consistent with the regional coherence (ReHo) studies of sensory-motor network (SMN) in ALS, which showed decreased regional brain coherence in the superior medial SMN and increased ReHo in the peripheral SMN areas (Zhou et al., 2014). Previous studies have put forth two hypotheses that may explain why the SMN of ALS patients is noted to be increasingly activated with elevated functional connectivity (Chio et al., 2014). Firstly, this heightened connectivity and activation in functionality may be a compensatory mechanism in response to structural damage, albeit not a permanent one as the disease progresses, with further neuro-degeneration limiting such a response. This could be strongly supported by the evidence that ALS patients with preserved diffusion tensor imaging (DTI) measures had been found to possess more widespread elevations in functional connectivity of sensorimotor networks compared to those with advanced corticospinal tract damage (Agosta et al., 2011). Secondly, neurodegeneration in ALS may lead to the loss of local inhibitory circuits, resulting in an heightened functional connectivity in these regions (Turner and Kiernan, 2012). This was supported by PET scan studies that found attenuated levels of GABA in the motor/premotor cortices of ALS patients (Lloyd et al., 2000; Foerster et al., 2012). In agreement with this second hypothesis, we also found increased functional connectivity between upper limb region of BA4 (Brainnetome #58) and the right caudate and bilateral thalamus in seed-based functional connectivity analysis (**Figure 2C**, dashed lines), which might cause symptoms of fasciculations, hyperreflexia, and spasticity in ALS patients clinically (Geevasinga et al., 2016).

### Changes of FCD in Extramotor Cortex

Although motor cortex impairment is the hallmark feature of ALS, there is increasing evidence indicating the widespread involvement of extra-motor brain areas. The temporal cortex exhibited extensive suppression in FCD, including the left superior temporal gyrus for short-range FCD and bilateral heschl gyrus, bilateral superior and middle temporal gyrus for long-range FCD, characterized by reduced functional connectivity distributed mainly in frontal, occipital, and parietal areas (**Figure 2B**). Our findings of temporal lobe involvement corresponds to existing neuro-imaging, neuro-psychological and neuro-pathological data. Firstly, findings of cortical thinning, reduced density and atrophy of adjacent white matter in temporal lobe were identified on neuroimaging studies performed with structural imaging techniques (Agosta et al., 2007; Mezzapesa et al., 2013; Menke et al., 2017). Widespread changes in functional connectivity were observed across the temporal–occipital cortex despite the absence of clinical behavioral symptoms (Loewe et al., 2017). Secondly, impairments in semantic fluency, a cognitive function under the control of the temporal lobe, has been documented in patients with ALS (Lepow et al., 2010). In addition, the grammatical deficits in ALS are associated with atrophy of the temporal regions and white matter projections in frontal–temporal network (Ash et al., 2015). Thirdly, pathological accumulations of the TDP-43 protein inclusions have been identified in approximately 40% of the temporal lobe in patients with ALS (Geser et al., 2008). Microglial activation as evidenced by significant binding of ^18^F-DPA-714 on PET imaging has been localized to the temporal cortices in the early stages of the disease (Corcia et al., 2012).

Both the bilateral rolandic operculum and right insula were also found to have decreased long-range FCD. The FCD reduction in bilateral rolandic operculum was consistent with previous MRS study that revealed significantly reduced NAA:Cr ratio in this region in ALS patients (Verma et al., 2013). A study that employed both voxel-wise meta-analysis and Jackknife sensitivity analysis concluded that the bilateral rolandic operculum indeed underwent a degree of gray matter atrophy (Shen et al., 2016). Given that rolandic operculum plays a key role in the neural pathways responsible for the output of articulated language (Eickhoff et al., 2006a,b), it is not unreasonable to postulate that this reduction in long-range FCD in this region might be responsible for dysarthria in ALS patients (Ash et al., 2015). The insula serves as a vital integration center for multiple senses that facilitates interpretation of signals related to motor control, visceral sensory perception, self-consciousness, and emotional regulation (Craig, 2002; Pollatos et al., 2007). Taken together, it can be inferred that dysfunctional long-range FCD in the insula may hint toward the presence of impaired motor monitoring, emotion and cognition in patients with ALS (Bungener et al., 2005; Mohammadi et al., 2015; Crespi et al., 2016).

The observed decreases of FCD also included the right lingual gyrus. ALS-related alterations within primary and associative visual areas have been observed before, including gray matter atrophy (Bede et al., 2013), cortical thinning (Mezzapesa et al., 2013), metabolic abnormalities (Verma et al., 2013; Pagani et al., 2014; Pagani et al., 2016), and reduced functional connectivity (Zhou et al., 2016). Functional damage to the visual cortices in ALS is further supported by findings of an impaired homotopic structural connectivity through corpus callosum subregion V, a network that is potentially mediated by temporo-occipital and parieto-occipital regions, thus further substantiating extra-motor involvement in ALS (Zhang et al., 2016).

The posterior and middle cingulum is the interconnected core region of the default mode network (DMN); (Raichle et al., 2001) that also connects to motor regions and includes extensive cortical area stream inputs through thalamus (Sudharshan et al., 2011). Patients with ALS were found to have attenuated long-range FCD in this region, characterized by impaired connections with frontal, temporal, occipital cortex, precuneus, basal ganglia and cerebellum (**Figure 2H**). Our result was in accordance with a previous fMRI study of reduced level of functional communication in DMN (Mohammadi et al., 2009) and a DTI study of impaired structural connectedness of the posterior cingulate regions to the motor network (Verstraete et al., 2011). In addition, reduced cingulate neuronal integrity has also been corroborated by several radiologic modalities, including DTI (Li et al., 2012), fMRI (Zhang et al., 2016; Zhou et al., 2016), MRS (Sudharshan et al., 2011), and arterial spin-labeling (ASL) MR imaging (Rule et al., 2010).

While the cerebellum is classically known for its integral functions in preserving intact motor abilities, emerging evidence over the past decade has alluded to its role in neuropsychiatric and cognitive processes (Stoodley and Schmahmann, 2009). We observed decreased FCD in the cerebellar vermis in ALS and decreased long-range functional connectivity between this region and the frontal cortex, basal ganglia, thalamus, and precuneus (**Figure 2E**). Previous neuroimaging studies have documented cerebellar degeneration in patients with ALS, as demonstrated by decreased gray matter volume and disturbed connectivity in its white matter tracts (Kassubek et al., 2005; Prell and Grosskreutz, 2013). Moreover, *post-hoc* covariance analysis has uncovered that while inferior cerebellar lobule atrophy mediates the motor symptoms in ALS, atrophy of the superior lobule and crus have been closely linked to the presence of neuropsychiatric and cognitive symptoms (Tan et al., 2014). However, some studies using PET techniques demonstrated hypermetabolism in the cerebellum in ALS patients and ascribed the relative hypermetabolism to a compensation for functional decline (Matias-Guiu et al., 2016; Buhour et al., 2017). We speculated that the discordance might be as a result of differences in methodology or patient cohorts with varying clinical and pathological characteristics.

Our results also revealed distinct patterns of increased FCD in left inferior parietal lobule/left angular gyrus (BA39, 40) in the patients group besides the other increased FCD cluster in premotor cortex discussed above. This area functions as the junction of the auditory, visual, and somatosensory cortexes, and is known to confer deficits in hand movements, visuomotor space, and shape representation frequently encountered in ALS (Phukan et al., 2007; Li et al., 2015). ALS related structural alterations within this region have been observed before, including volume atrophy, cortical thinning, and surface area reduction (Grosskreutz et al., 2006; Verstraete et al., 2012; Thorns et al., 2013). Functional MRI studies have revealed elevated functional connectivity and ReHo in the inferior parietal lobule that are in accordance with our findings (Zhou et al., 2014; Loewe et al., 2017). Furthermore, the increase in functional connectivity can be interpreted as a mixture of compensatory mechanisms counteracting the evolving structural lesion and transcallosal disinhibition (Loewe et al., 2017).

### Partial Correlation Analysis and GSR Effect

In this paper, no statistical correlation was found between aberrant FCDs and clinical variables after Bonferroni correction in the partial correlation analysis. This might suggest that aberrant FCD was an independent characteristic of ALS disease. However, it is possible that the modest sample size of our patient cohort precluded the statistical power needed to produce significant results. Additional behavioral and neuropsychology assessments of ALS patients would be helpful for a better interpretation and exploration of brain–behavior relationships.

How GSR affected the comparisons of short- and long-range FCD between HCs and ALS patients were also studied since GSR is commonly utilized to control scanner instabilities in resting functional connectivity analysis. On the other hand, fluctuations of global signal could represent true electrophysiological activity (Scholvinck et al., 2010) and GSR may inadvertently cause spurious anti-correlations (Murphy et al., 2009; Saad et al., 2012). We found additional group differences of FCD in thalamus and medial superior frontal gyrus in the GSR condition, and it was similar with results about GSR effect on FCD in Tomasi’s studies that GSR would cause spuriously increases in FCD in white matter and subcortical regions including thalamus (Tomasi et al., 2016a,b).

## Limitations

One caveat is that we utilized the AlphaSim program, which possesses a relatively weak correction strategy when utilized for multiple comparisons in cohorts of small sample sizes. Therefore, the significance of these preliminary findings would be enhanced in replicated studies on larger samples sizes. Secondly, the modest number of patients precluded the statistical power necessary for further sub-group analysis (e.g., upper motor neuron-predominant group vs. lower motor neuron-predominant group), thus barring us from being able to totally exclude the influence of clinicopathological heterogeneity. A more homogenous sample would allow for this effect to be negated in future studies. Thirdly, more behavior or neuropsychology assessments of ALS patients will allow us to further explore brain-behavior relationships. Lastly, only a single threshold was selected for FCD map calculation, given that this was taken to be an exploratory study. Further studies will benefit from incorporating a range of thresholds in order to better examine results stability.

## Conclusion

In conclusion, our study investigated the changes of brain FCD in ALS patients. We found decreased FCD in primary motor cortex in the patients group compared with HCs. In the meantime, we observed increased FCD in premotor cortex and the peripheral inferior parietal lobe. We documented that the increased FCD could be interpreted as a mixture of compensatory mechanisms and transcallosal disinhibition. Moreover, the increased functional connectivity between premotor cortex and caudate as well as thalamus might reflect the disturbance of the local inhibitory circuitry. Furthermore, our study also showed multiple extra-motor brain regions with decreased FCD including temporal cortex, insula, cingulate gyrus, and the occipital cortex, which might underlie the motor control, language, visuoperceptual, and high order cognition deficits in ALS and add the evidence that ALS is a multisystem disorder. This study improves our understanding of the neural mechanisms underlying ALS.

## Author Contributions

WL: formulation of project idea and execution and authoring of first draft. JZ: formulation of project idea, review and critique of article. CZ: acquisition of MRI data. WH: analysis of MRI data. JH: patient enrollment and acquisition of clinical data. HF: formulation of project idea, review and critique of article. XZ: formulation of project idea, review and critique of article.

## Conflict of Interest Statement

The authors declare that the research was conducted in the absence of any commercial or financial relationships that could be construed as a potential conflict of interest.

## Footnotes

1 http://www.fil.ion.ucl.ac.uk/spm

2 http://www.neuro.uestc.edu.cn/NIT.html

3 http://restfmri.net/forum/index.php?q=rest

4 http://atlas.brainnetome.org
