# Supplementary material for: Abnormal Functional Connectivity Density in Amyotrophic Lateral Sclerosis
Source: Front Aging Neurosci. 2018 Jul 17;10:215. doi: 10.3389/fnagi.2018.00215 (PMC6056617; doi:10.3389/fnagi.2018.00215)
Supplement: Supplementary file 1 [file Data_Sheet_1.docx]

Supplementary Material

**Abnormal Functional Connectivity Density in Amyotrophic Lateral Sclerosis**

Weina Li, Jiuquan Zhang^*^, Chaoyang Zhou, Wensheng Hou, Jun Hu, Hua Feng, Xiaolin Zheng^*^

**Correspondence:** Xiaolin Zheng, [zxl@cqu.edu.cn](mailto:zxl@cqu.edu.cn) ;

Jiuquan Zhang, [zhangjq_radiol@foxmail.com](mailto:zhangjq_radiol@foxmail.com).

# 1. Supplementary Figures


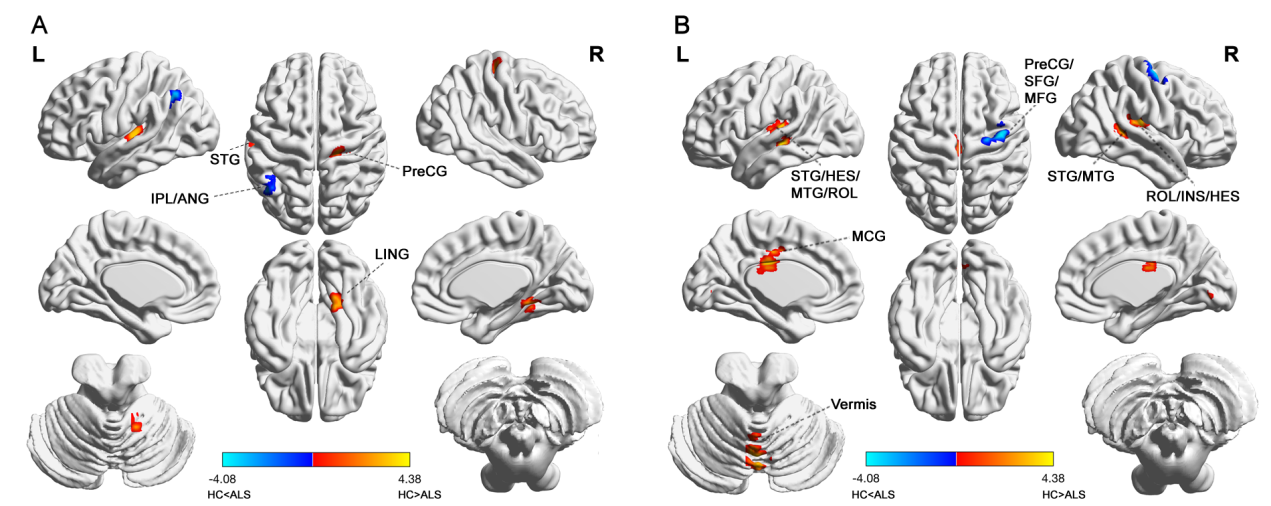


**Figure S1.** Distribution of statistical differences in short-range and long-range FCDs between ALS patients and healthy controls (HCs) considering the confounding effects of micro head movement parameter (mFD). Voxel-wise two-sample t-test with four covariates (age, gender, total intracranial volume, micro head movement parameter (mFD)) was used to contrast short-range and long-range FCD maps across groups (p<0.05, AlphaSim corrected) in no global signal regression condition. **(A), (B)** shows the distribution of short-range FCD and long-range FCD differences between the two groups respectively, with warm color representing FCD value of HC lager than ALS and cold color denoting FCD value of HC smaller than ALS. ANG, angular gyrus; HES, heschl gyrus; INS, insula; IPL, inferior parietal; LING, lingual gyrus; MCG, middle cingulate; MFG, middle frontal gyrus; MTG, middle temporal gyrus; PreCG, precental gyrus; ROL, rolandic operculum; SFG, superior frontal gyrus; STG, superior temporal gyrus; L, left; R, right.


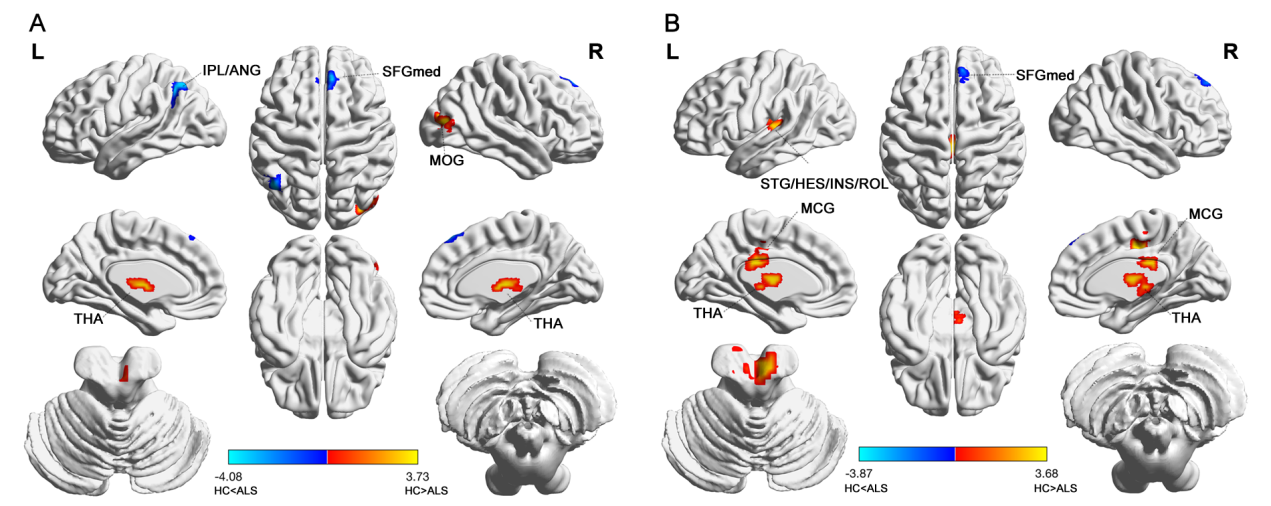


**Figure S2.** Distribution of statistical differences in short-range and long-range FCDs between ALS patients and healthy controls (HCs) in global signal regression condition. Voxel-wise two-sample t-test with three covariates (age, gender, total intracranial volume) was used to contrast short-range and long-range FCD maps across groups (p<0.05, AlphaSim corrected) in global signal regression condition. **(A), (B)** shows the distribution of short-range FCD and long-range FCD differences between the two groups respectively, with warm color representing FCD value of HC lager than ALS and cold color denoting FCD value of HC smaller than ALS. ANG, angular gyrus; HES, heschl gyrus; INS, insula; IPL, inferior parietal; MCG, middle cingulate; MOG, middle occipital gyrus; ROL, rolandic operculum; SFGmed, superior frontal gyrus (medial); STG, superior temporal gyrus; THA, thalamus; L, left; R, right.

# 2. Supplementary Tables

## Table S1. Brainnetome Atlas (<http://atlas.brainnetome.org/>)

| **Lobe** | **Gyrus** | **Label ID** | **Label name** | **Descriptions** |
| --- | --- | --- | --- | --- |
| **Frontal Lobe** | SFG, Superior Frontal Gyrus | 1,2 | A8m | A8m, medial area 8 |
|  |  | 3,4 | A8dl | A8dl, dorsolateral area 8 |
|  |  | 5,6 | A9l | A9l, lateral area 9 |
|  |  | 7,8 | A6dl | A6dl, dorsolateral area 6 |
|  |  | 9,10 | A6m | A6m, medial area 6 |
|  |  | 11,12 | A9m | A9m,medial area 9 |
|  |  | 13,14 | A10m | A10m, medial area 10 |
|  | MFG, Middle Frontal Gyrus | 15,16 | A9/46d | A9/46d, dorsal area 9/46 |
|  |  | 17,18 | IFJ | IFJ, inferior frontal junction |
|  |  | 19,20 | A46 | A46, area 46 |
|  |  | 21,22 | A9/46v | A9/46v, ventral area 9/46 |
|  |  | 23,24 | A8vl | A8vl, ventrolateral area 8 |
|  |  | 25,26 | A6vl | A6vl, ventrolateral area 6 |
|  |  | 27,28 | A10l | A10l, lateral area10 |
|  | IFG, Inferior Frontal Gyrus | 29,30 | A44d | A44d,dorsal area 44 |
|  |  | 31,32 | IFS | IFS, inferior frontal sulcus |
|  |  | 33,34 | A45c | A45c, caudal area 45 |
|  |  | 35,36 | A45r | A45r, rostral area 45 |
|  |  | 37,38 | A44op | A44op, opercular area 44 |
|  |  | 39,40 | A44v | A44v, ventral area 44 |
|  | OrG, Orbital Gyrus | 41,42 | A14m | A14m, medial area 14 |
|  |  | 43,44 | A12/47o | A12/47o, orbital area 12/47 |
|  |  | 45,46 | A11l | A11l, lateral area 11 |
|  |  | 47,48 | A11m | A11m, medial area 11 |
|  |  | 49,50 | A13 | A13, area 13 |
|  |  | 51,52 | A12/47l | A12/47l, lateral area 12/47 |
|  | PrG, Precentral Gyrus | 53,54 | A4hf | A4hf, area 4(head and face region) |
|  |  | 55,56 | A6cdl | A6cdl, caudal dorsolateral area 6 |
|  |  | 57,58 | A4ul | A4ul, area 4(upper limb region) |
|  |  | 59,60 | A4t | A4t, area 4(trunk region) |
|  |  | 61,62 | A4tl | A4tl, area 4(tongue and larynx region) |
|  |  | 63,64 | A6cvl | A6cvl, caudal ventrolateral area 6 |
|  | PCL, Paracentral Lobule | 65,66 | A1/2/3ll | A1/2/3ll, area1/2/3 (lower limb region) |
|  |  | 67,68 | A4ll | A4ll, area 4, (lower limb region) |
| **Temporal Lobe** | STG, Superior Temporal Gyrus | 69,70 | A38m | A38m, medial area 38 |
|  |  | 71,72 | A41/42 | A41/42, area 41/42 |
|  |  | 73,74 | TE1.0 and TE1.2 | TE1.0 and TE1.2 |
|  |  | 75,76 | A22c | A22c, caudal area 22 |
|  |  | 77,78 | A38l | A38l, lateral area 38 |
|  |  | 79,80 | A22r | A22r, rostral area 22 |
|  | MTG, Middle Temporal Gyrus | 81,82 | A21c | A21c, caudal area 21 |
|  |  | 83,84 | A21r | A21r, rostral area 21 |
|  |  | 85,86 | A37dl | A37dl, dorsolateral area37 |
|  |  | 87,88 | aSTS | aSTS, anterior superior temporal sulcus |
|  | ITG, Inferior Temporal Gyrus | 89,90 | A20iv | A20iv, intermediate ventral area 20 |
|  |  | 91,92 | A37elv | A37elv, extreme lateroventral area37 |
|  |  | 93,94 | A20r | A20r, rostral area 20 |
|  |  | 95,96 | A20il | A20il, intermediate lateral area 20 |
|  |  | 97,98 | A37vl | A37vl, ventrolateral area 37 |
|  |  | 99,100 | A20cl | A20cl, caudolateral of area 20 |
|  |  | 101,102 | A20cv | A20cv, caudoventral of area 20 |
|  | FuG, Fusiform Gyrus | 103,104 | A20rv | A20rv, rostroventral area 20 |
|  |  | 105,106 | A37mv | A37mv, medioventral area37 |
|  |  | 107,108 | A37lv | A37lv, lateroventral area37 |
|  | PhG, Parahippocampal Gyrus | 109,110 | A35/36r | A35/36r, rostral area 35/36 |
|  |  | 111,112 | A35/36c | A35/36c, caudal area 35/36 |
|  |  | 113,114 | TL | TL, area TL (lateral PPHC, posterior parahippocampal gyrus) |
|  |  | 115,116 | A28/34 | A28/34, area 28/34 (EC, entorhinal cortex) |
|  |  | 117,118 | TI | TI, area TI(temporal agranular insular cortex) |
|  |  | 119,120 | TH | TH, area TH (medial PPHC) |
|  | pSTS, posterior Superior Temporal Sulcus | 121,122 | rpSTS | rpSTS, rostroposterior superior temporal sulcus |
|  |  | 123,124 | cpSTS | cpSTS, caudoposterior superior temporal sulcus |
| **Parietal Lobe** | SPL, Superior Parietal Lobule | 125,126 | A7r | A7r, rostral area 7 |
|  |  | 127,128 | A7c | A7c, caudal area 7 |
|  |  | 129,130 | A5l | A5l, lateral area 5 |
|  |  | 131,132 | A7pc | A7pc, postcentral area 7 |
|  |  | 133,134 | A7ip | A7ip, intraparietal area 7(hIP3) |
|  | IPL, Inferior Parietal Lobule | 135,136 | A39c | A39c, caudal area 39(PGp) |
|  |  | 137,138 | A39rd | A39rd, rostrodorsal area 39(Hip3) |
|  |  | 139,140 | A40rd | A40rd, rostrodorsal area 40(PFt) |
|  |  | 141,142 | A40c | A40c, caudal area 40(PFm) |
|  |  | 143,144 | A39rv | A39rv, rostroventral area 39(PGa) |
|  |  | 145,146 | A40rv | A40rv, rostroventral area 40(PFop) |
|  | PCun, Precuneus | 147,148 | A7m | A7m, medial area 7(PEp) |
|  |  | 149,150 | A5m | A5m, medial area 5(PEm) |
|  |  | 151,152 | dmPOS | dmPOS, dorsomedial parietooccipital sulcus(PEr) |
|  |  | 153,154 | A31 | A31, area 31 (Lc1) |
|  | PoG, Postcentral Gyrus | 155,156 | A1/2/3ulhf | A1/2/3ulhf, area 1/2/3(upper limb, head and face region) |
|  |  | 157,158 | A1/2/3tonIa | A1/2/3tonIa, area 1/2/3(tongue and larynx region) |
|  |  | 159,160 | A2 | A2, area 2 |
|  |  | 161,162 | A1/2/3tru | A1/2/3tru, area1/2/3(trunk region) |
| **Insular Lobe** | INS, Insular Gyrus | 163,164 | G | G, hypergranular insula |
|  |  | 165,166 | vIa | vIa, ventral agranular insula |
|  |  | 167,168 | dIa | dIa, dorsal agranular insula |
|  |  | 169,170 | vId/vIg | vId/vIg, ventral dysgranular and granular insula |
|  |  | 171,172 | dIg | dIg, dorsal granular insula |
|  |  | 173,174 | dId | dId, dorsal dysgranular insula |
| **Limbic Lobe** | CG, Cingulate Gyrus | 175,176 | A23d | A23d, dorsal area 23 |
|  |  | 177,178 | A24rv | A24rv, rostroventral area 24 |
|  |  | 179,180 | A32p | A32p, pregenual area 32 |
|  |  | 181,182 | A23v | A23v, ventral area 23 |
|  |  | 183,184 | A24cd | A24cd, caudodorsal area 24 |
|  |  | 185,186 | A23c | A23c, caudal area 23 |
|  |  | 187,188 | A32sg | A32sg, subgenual area 32 |
| **Occipital Lobe** | MVOcC, MedioVentral Occipital Cortex | 189,190 | cLinG | cLinG, caudal lingual gyrus |
|  |  | 191,192 | rCunG | rCunG, rostral cuneus gyrus |
|  |  | 193,194 | cCunG | cCunG, caudal cuneus gyrus |
|  |  | 195,196 | rLinG | rLinG, rostral lingual gyrus |
|  |  | 197,198 | vmPOS | vmPOS,ventromedial parietooccipital sulcus |
|  | LOcC, lateral Occipital Cortex | 199,200 | mOccG | mOccG, middle occipital gyrus |
|  |  | 201,202 | V5/MT+ | V5/MT+, area V5/MT+ |
|  |  | 203,204 | OPC | OPC, occipital polar cortex |
|  |  | 205,206 | iOccG | iOccG, inferior occipital gyrus |
|  |  | 207,208 | msOccG | msOccG, medial superior occipital gyrus |
|  |  | 209,210 | lsOccG | lsOccG, lateral superior occipital gyrus |
| **Subcortical Nuclei** | Amyg, Amygdala | 211,212 | mAmyg | mAmyg, medial amygdala |
|  |  | 213,214 | lAmyg | lAmyg, lateral amygdala |
|  | Hipp, Hippocampus | 215,216 | rHipp | rHipp, rostral hippocampus |
|  |  | 217,218 | cHipp | cHipp, caudal hippocampus |
|  | BG, Basal Ganglia | 219,220 | vCa | vCa, ventral caudate |
|  |  | 221,222 | GP | GP, globus pallidus |
|  |  | 223,224 | NAC | NAC, nucleus accumbens |
|  |  | 225,226 | vmPu | vmPu, ventromedial putamen |
|  |  | 227,228 | dCa | dCa, dorsal caudate |
|  |  | 229,230 | dlPu | dlPu, dorsolateral putamen |
|  | Tha, Thalamus | 231,232 | mPFtha | mPFtha, medial pre-frontal thalamus |
|  |  | 233,234 | mPMtha | mPMtha, pre-motor thalamus |
|  |  | 235,236 | Stha | Stha, sensory thalamus |
|  |  | 237,238 | rTtha | rTtha, rostral temporal thalamus |
|  |  | 239,240 | PPtha | PPtha, posterior parietal thalamus |
|  |  | 241,242 | Otha | Otha, occipital thalamus |
|  |  | 243,244 | cTtha | cTtha, caudal temporal thalamus |
|  |  | 245,246 | lPFtha | lPFtha, lateral pre-frontal thalamus |
| Cerebellum | Cerebellum | 247 | L_I-IV | Cerebellum_Left_I-IV |
|  |  | 248 | R_I-IV | Cerebellum_Right_I-IV |
|  |  | 249 | L_V | Cerebellum_Left_V |
|  |  | 250 | R_V | Cerebellum_Right_V |
|  |  | 251 | L_VI | Cerebellum_Left_VI |
|  |  | 252 | V_VI | Cerebellum_Vermis_VI |
|  |  | 253 | R_VI | Cerebellum_Right_VI |
|  |  | 254 | L_Crus_I | Cerebellum_Left_Crus_I |
|  |  | 255 | V_Crus_I | Cerebellum_Vermis_Crus_I |
|  |  | 256 | R_Crus_I | Cerebellum_Right_Crus_I |
|  |  | 257 | L_Crus_II | Cerebellum_Left_Crus_II |
|  |  | 258 | V_Crus_II | Cerebellum_Vermis_Crus_II |
|  |  | 259 | R_Crus_II | Cerebellum_Right_Crus_II |
|  |  | 260 | L_VIIb | Cerebellum_Left_VIIb |
|  |  | 261 | V_VIIb | Cerebellum_Vermis_VIIb |
|  |  | 262 | R_VIIb | Cerebellum_Right_VIIb |
|  |  | 263 | L_VIIIa | Cerebellum_Left_VIIIa |
|  |  | 264 | V_VIIIa | Cerebellum_Vermis_VIIIa |
|  |  | 265 | R_VIIIa | Cerebellum_Right_VIIIa |
|  |  | 266 | L_VIIIb | Cerebellum_Left_VIIIb |
|  |  | 267 | V_VIIIb | Cerebellum_Vermis_VIIIb |
|  |  | 268 | R_VIIIb | Cerebellum_Right_VIIIb |
|  |  | 269 | L_IX | Cerebellum_Left_IX |
|  |  | 270 | V_IX | Cerebellum_Vermis_IX |
|  |  | 271 | R_IX | Cerebellum_Right_IX |
|  |  | 272 | L_X | Cerebellum_Left_X |
|  |  | 273 | V_X | Cerebellum_Vermis_X |
|  |  | 274 | R_X | Cerebellum_Right_X |

## Table S2. Short- and long-range FCD alterations in ALS patients compared with healthy controls in global signal regression (GSR) condition

| **FCD** | **Brain Regions** | **Brodmann's Area** | **Brainnetome Atlas Subregion^b^** | **Cluster Size (voxel)^a^** | **Peak MNI Coordinate (x,y,z)** | **Peak Intensity (T value)** |
| --- | --- | --- | --- | --- | --- | --- |
| **Short-range FCD** | ***HC>ALS*** |  |  |  |  |  |
|  | Right Middle occipital gyrus | 19 | mOccG(Brainnetome #200) | 60 | 39,-75,9 | 3.7336 |
|  | Bilateral Thalamus | / | rTtha(Brainnetome #238/237) | 39 | 3,-12,6 | 3.6454 |
|  | ***HC<ALS*** |  |  |  |  |  |
|  | Right Medial superior frontal gyrus | 8 | A9l(Brainnetome #6) | 58 | 9,42,54 | -4.0792 |
|  | Left Inferior parietal lobule, Left Angular gyurs | 40,39 | A40c(Brainnetome #141), A39rd(Brainnetome #137) | 70 | -42,-51,39 | -3.5308 |
| **Long-range FCD** | ***HC>ALS*** |  |  |  |  |  |
|  | Bilateral Thalamus | / | rTtha(Brainnetome #231/238/237) | 87 | -3,-12,9 | 3.3433 |
|  | Left Superior temporal gyrus, Left Heschl gyrus, Left Rolandic operculum, Left Insula | 41,13 | TE1.0 and TE1.2(Brainnetome #73), A41/42(Brainnetome #71),G (Brainnetome #163) | 96 | -39,-33,6 | 3.6089 |
|  | Left median cingulate gyrus | 23 | A23d (Brainnetome #175) | 63 | -6,-27,27 | 3.6 |
|  | Right median cingulate gyrus | 23 | A23c (Brainnetome #186) | 55 | 15,-12,42 | 3.6757 |
|  | ***HC<ALS*** |  |  |  |  |  |
|  | Right Medial superior frontal gyrus | 8 | A9l(Brainnetome #6) | 53 | 9,45,48 | -3.8687 |

^a^Statistical significance was set at a voxel-wise p < 0.01, in conjunction with cluster wise AlphaSim (rmm = 5, clusters = 36) to correct for multiple comparisons. ^b^Brainnetome Atlas, a brain atlas based on connectional architecture, http://atlas.brainnetome.org/
